# 

**Published:** 1919-12

**Authors:** 


### ANNOUNCEMENT

### American Institute of Dental Teachers

The next annual meeting of the American Institute of Dental Teachers will be held at Detroit, Michigan, January 27-29, 1920. Hotel Statler will be the headquarters.

A cordial invitation is extended to all persons interested in dental teaching.—Dr. Russell W. Bunting, President, Dr. Abram Hoffman, Secretary, 381 Linwood Ave., Buffalo, N. Y.

